# Supplementary material for: Tead1a Initiates Transcriptional Priming Through the TEAD1a/YAP‐Notch1‐Spi1/Cebpα Axis to Promote Neutrophil Fate
Source: Adv Sci (Weinh). 2025 Aug 28;12(41):e05441. doi: 10.1002/advs.202505441 (PMC12591127; doi:10.1002/advs.202505441)
Supplement: Supplementary file 4 — Supplementary Dataset [file ADVS-12-e05441-s004.zip › 3-1.zip!/3-1/02-1.cluster/cluster2/cluster2_vlnplot.pdf]

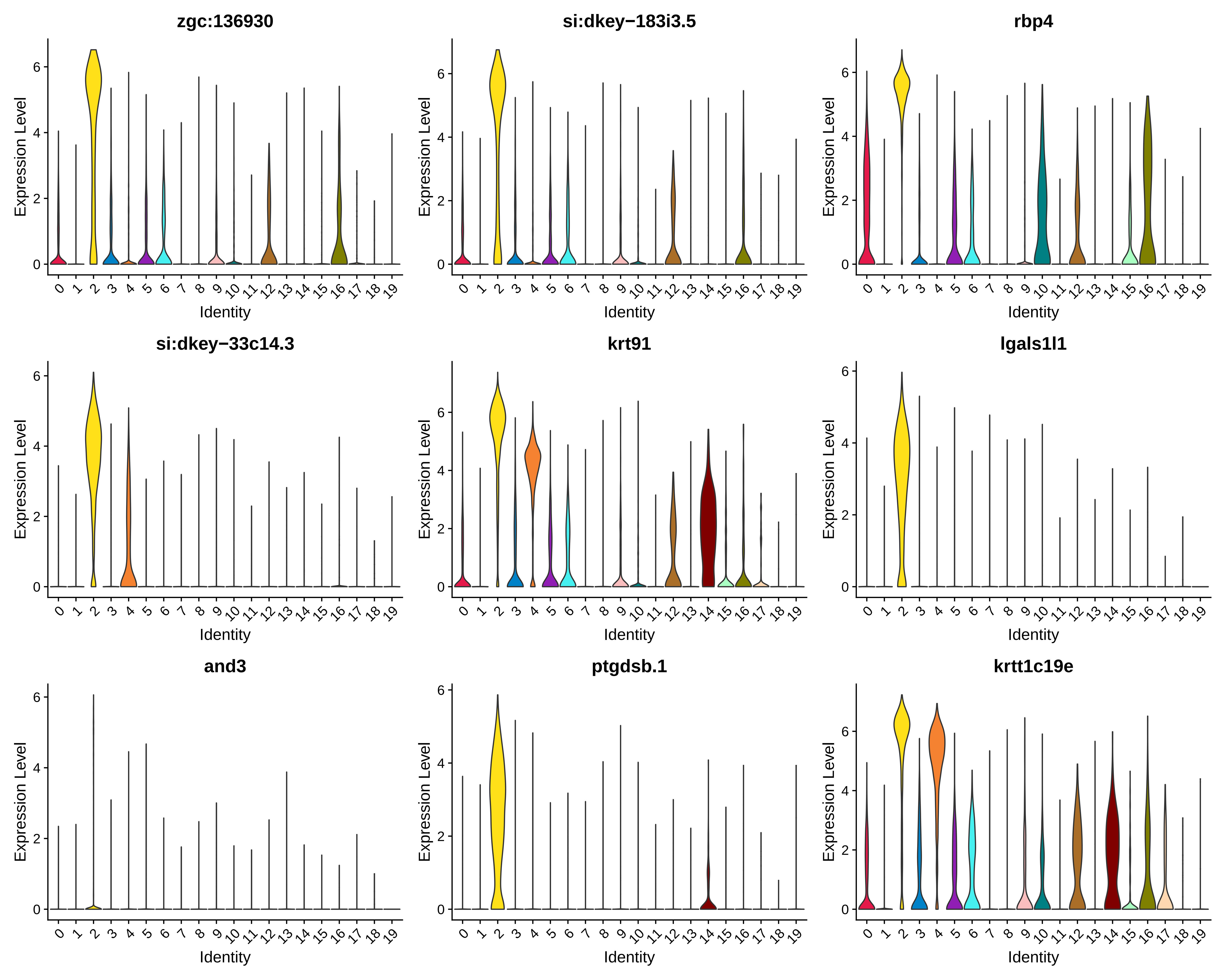
zgc:136930
si:dkey−183i3.5
rbp4
si:dkey−33c14.3
krt91
lgals1l1
and3
ptgdsb.1
krtt1c19e
Expression Level
Identity
